# Increases in plasma sheet temperature with solar wind driving during substorm growth phases

**DOI:** 10.1002/2014GL062400

**Published:** 2014-12-23

**Authors:** C Forsyth, C E J Watt, I J Rae, A N Fazakerley, N M E Kalmoni, M P Freeman, P D Boakes, R Nakamura, I Dandouras, L M Kistler, C M Jackman, J C Coxon, C M Carr

**Affiliations:** 1 UCL Mullard Space Science LaboratoryDorking, UK; 2 Department of Meteorology, University of ReadingReading, UK; 3 British Antarctic SurveyCambridge, UK; 4 Space Research Institute, Austrian Academy of SciencesGraz, Austria; 5 Institut de Recherche en Astrophysique et Planétologie, University of ToulouseToulouse, France; 6 CNRS, IRAPToulouse, France; 7 Space Science Centre, Morse Hall, University of New HampshireDurham, New Hampshire, USA; 8 School of Physics & Astronomy, University of SouthamptonSouthampton, UK; 9 Department Physics and Astronomy, University of LeicesterLeicester, UK; 10 Department of Physics, Imperial College LondonLondon, UK

**Keywords:** substorm, growth phase, plasma sheet, temperature

## Abstract

During substorm growth phases, magnetic reconnection at the magnetopause extracts ∼10^15^ J from the solar wind which is then stored in the magnetotail lobes. Plasma sheet pressure increases to balance magnetic flux density increases in the lobes. Here we examine plasma sheet pressure, density, and temperature during substorm growth phases using 9 years of Cluster data (>316,000 data points). We show that plasma sheet pressure and temperature are higher during growth phases with higher solar wind driving, whereas the density is approximately constant. We also show a weak correlation between plasma sheet temperature before onset and the minimum SuperMAG AL (SML) auroral index in the subsequent substorm. We discuss how energization of the plasma sheet before onset may result from thermodynamically adiabatic processes; how hotter plasma sheets may result in magnetotail instabilities, and how this relates to the onset and size of the subsequent substorm expansion phase.

## 1. Introduction

Magnetic reconnection at the magnetopause is estimated to extract ∼10^15^ J of energy from the solar wind during the substorm cycle [*Tanskanen et al.,* 2002]. This energy is stored as magnetic energy in the magnetotail lobes until released by reconnection in the magnetotail, being roughly equally partitioned between enhanced particle precipitation and Joule heating in the ionosphere; energization of the ring current; and loss in plasmoids and the postplasmoid plasma sheet [*Richardson et al.,* 1987; *Ieda et al.,* 1998]. The timing of release, the amount of available energy released, how and why it is partitioned into different energy forms and pathways, and the factors controlling these properties are significant unknowns in our understanding of substorm dynamics [*Koskinen and Tanskanen*, 2002; *Freeman and Morley*, 2004; *Morley et al.,* 2007].

As magnetopause reconnection occurs, the dayside magnetosphere is eroded, moving inward by 10–20% [*Aubry*, 1970; *Holzer and Slavin,* 1978; *Sibeck et al.,* 1991; *Shue et al.,* 1997; *Volwerk et al.,* 2011], the cusps move equatorward and the magnetotail expands to accommodate the increasing open magnetic flux created by the reconnection. The increase in flaring of the near-Earth magnetotail increases the solar wind ram pressure transmitted through the magnetopause, which is balanced by an increase in the total pressure exerted from within by the increased magnetic flux in the lobes [*Coroniti and Kennel*, 1972; *Shue et al.,* 1997]. Theoretical models of these magnetospheric changes during the growth phase have been validated experimentally for a few events [e.g., *McPherron et al.,* 1973; *Fairfield et al.,* 1981; *Kistler et al.,* 1993].

The total pressure of the plasma sheet approximately balances the lobe pressure. As such, increases in the lobe magnetic pressure during substorm growth phases result in changes in the plasma sheet density and temperature. *Nagai et al.* [1997] and *Kistler et al.* [2006] found that during substorm growth phases ion density increased while temperature was unchanged, implying that the plasma sheet acts isothermally. This contrasts studies showing that the plasma sheet is thermodynamically adiabatic [e.g., *Baumjohann and Paschmann,* 1989; *Goertz and Baumjohann,* 1991] and the statistical analysis of plasma sheet density by *Nagata et al.* [2008] that showed only a small difference in plasma sheet density under long periods of northward or southward interplanetary magnetic field (IMF). This apparent discrepancy is significant since a number of plasma instabilities that may be responsible for substorm onset depend on plasma sheet density and temperature [e.g., *Lui*, 2004]. Thus, in order to understand the potential mechanisms for substorm onset, we must understand the variations in plasma sheet properties before onset.

Using 9 years of Cluster data, we statistically examine how the plasma and magnetic field in the magnetotail vary with solar wind energy input during substorm growth phases. Using new advances in the identification of magnetotail regions [*Boakes et al.,* 2014], we separate observations from the lobes and plasma sheet. The solar wind energy input is derived from time averaged upstream measurements of the solar wind plasma and field parameters (OMNI—http://nssdcftp.gsfc.nasa.gov/spacecraft_data/omni/).

## 2. Data

Between 2001 and 2009, the Cluster spacecraft orbited the Earth in a near-polar orbit, with an apogee of ∼19 *R_E_* in the magnetotail during the Northern Hemisphere summer. During each orbit, the spacecraft passed through the northern lobes, plasma sheet, and southern lobes, with the orbits sweeping from dawn to dusk each year.

Using data from the Cluster Ion Spectrometer Composition Distribution Function sensor (CIS-CODIF) [*Reme et al.,* 1997] and Fluxgate Magnetometer (FGM) [*Balogh et al.,* 1997] instruments on Cluster 4, we examine the ion plasma and magnetic field in the magnetotail between 2001 and 2009. We use data from the Cluster 4 CIS-CODIF sensor which remained operational throughout this period, to avoid any cross-calibration issues arising from using different instruments. We examine the midtail region, defined here as *X* < −10 *R_E_* and |*Y* | < 5 *R_E_*.

In order to identify growth phase intervals, we isolated those times that were not during substorm expansion or recovery phases using a technique similar to *Juusola et al.* [2011] but applied to the SuperMAG AL (SML) auroral index [*Newell and Gjerloev,* 2011a, 2011b; *Gjerloev*, 2012] rather than AL. Changes in SML (dSML/d*t*) were calculated between 2001 and 2010 and filtered using a 60 min low-pass filter to remove short period variations. The median positive and negative changes were then calculated. Intervals during which dSML/d*t* was less (more) than the median negative (positive) change were labeled as expansion (recovery) phase times. The main difference between our method and that of *Juusola et al.* [2011] is that we take all nonexpansion or recovery phase intervals to be growth phases (*Juusola et al.* [2011] only considered southward IMF intervals) since solar wind power input functions, such as the *ε* function [*Perreault and Akasofu,* 1978; *Akasofu*, 1979; *Morley et al.,* 2007], are nonzero for all but purely northward IMF.

For each Cluster data point, we calculate the mean solar wind power input over the preceding 15 min using the 1 min resolution OMNI data and the function 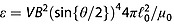 where *V* is the solar wind speed, *B* is the interplanetary magnetic field (IMF) strength, *θ* is the clock angle of the IMF with respect to the Earth's magnetic dipole moment, and *ℓ*_0_ is a scaling constant equal to 7 *R_E_*.

**Figure 1:**
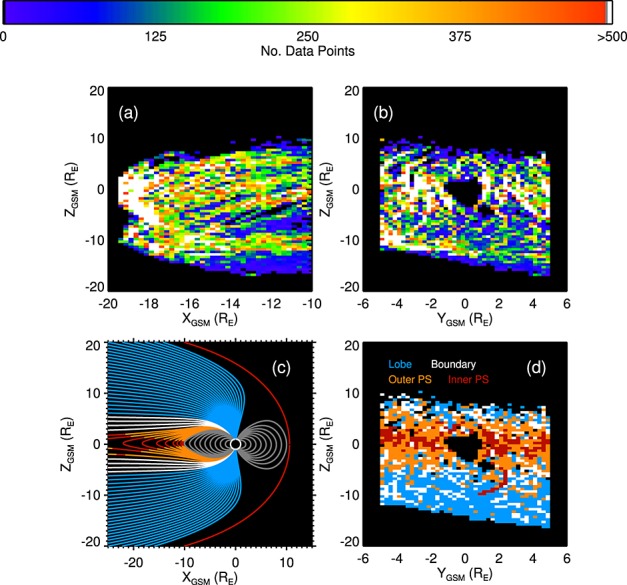
Cluster 4's coverage of the magnetotail. (a) The number of data points binned into 0.25 *R_E_* bins in *X* and *Y* and 0.5 *R_E_* bins in *Z* projected onto (a) the *XZ* GSM plane; (b) the *XY* GSM plane. (c) *Tsyganenko and Stern* [1996] model magnetic field lines color coded by magnetotail regions (see Figure 1d for color key) projected onto the *XZ* GSM plane. (d) The modal region encountered by Cluster 4 projected onto the *YZ* plane.

Figure 1 shows the data coverage from Cluster 4 projected onto the (Figure 1a) *XZ* and (Figure 1b) *XY* GSM planes. Magnetotail regions are identified from the European Cluster Assimilation Technology (ECLAT) database [*Boakes et al.,* 2014]. These are defined as the lobes, a boundary region, outer plasma sheet, inner plasma sheet. Utilizing Cluster's unique four spacecraft configuration and the curlometer technique [*Dunlop et al.,* 1988], threshold values in plasma *β* were determined for the different regions based on statistical relations between *β* and magnetotail currents (except for the inner plasma sheet, which has a threshold criteria in the magnetic field *B_xy_* component). These criteria compared well with other methods of region determination [*Boakes et al.,* 2014]. Figure 1c shows a simple model of the approximate locations of the ECLAT regions with their corresponding field lines in the *XZ* plane and Figure 1d shows the modal ECLAT regions encountered by Cluster 4 projected onto the *XY* GSM plane.

Cluster provides good coverage of the lobes and plasma sheet, with a median sample density of 1376 data points per *R_E_*^2^ in the *XY* plane (quartiles of 552 and 2239 *R_E_*^−2^) and 1728 *R_E_*^−2^ in the *XZ* plane (quartiles of 728 and 2944 *R_E_*^−2^). Figures 1b and 1d show there was greater spatial coverage of the lobes but greater temporal coverage of the plasma sheet, with 61,992 and 254,652 data points, respectively. The ECLAT region determination for Cluster 4 became less reliable after 2003 due to the degradation of the CIS-CODIF sensor, particularly the boundary region and outer plasma sheet [*Boakes et al.,* 2014]. However, overall Figure 1d shows the occurrence of the plasma sheet and lobes in the locations we expect. We limit the effects of any region misidentification by combining data from the boundary region, outer plasma sheet, and inner plasma sheet to show trends in the whole plasma sheet.

## 3. Results

**Figure 2:**
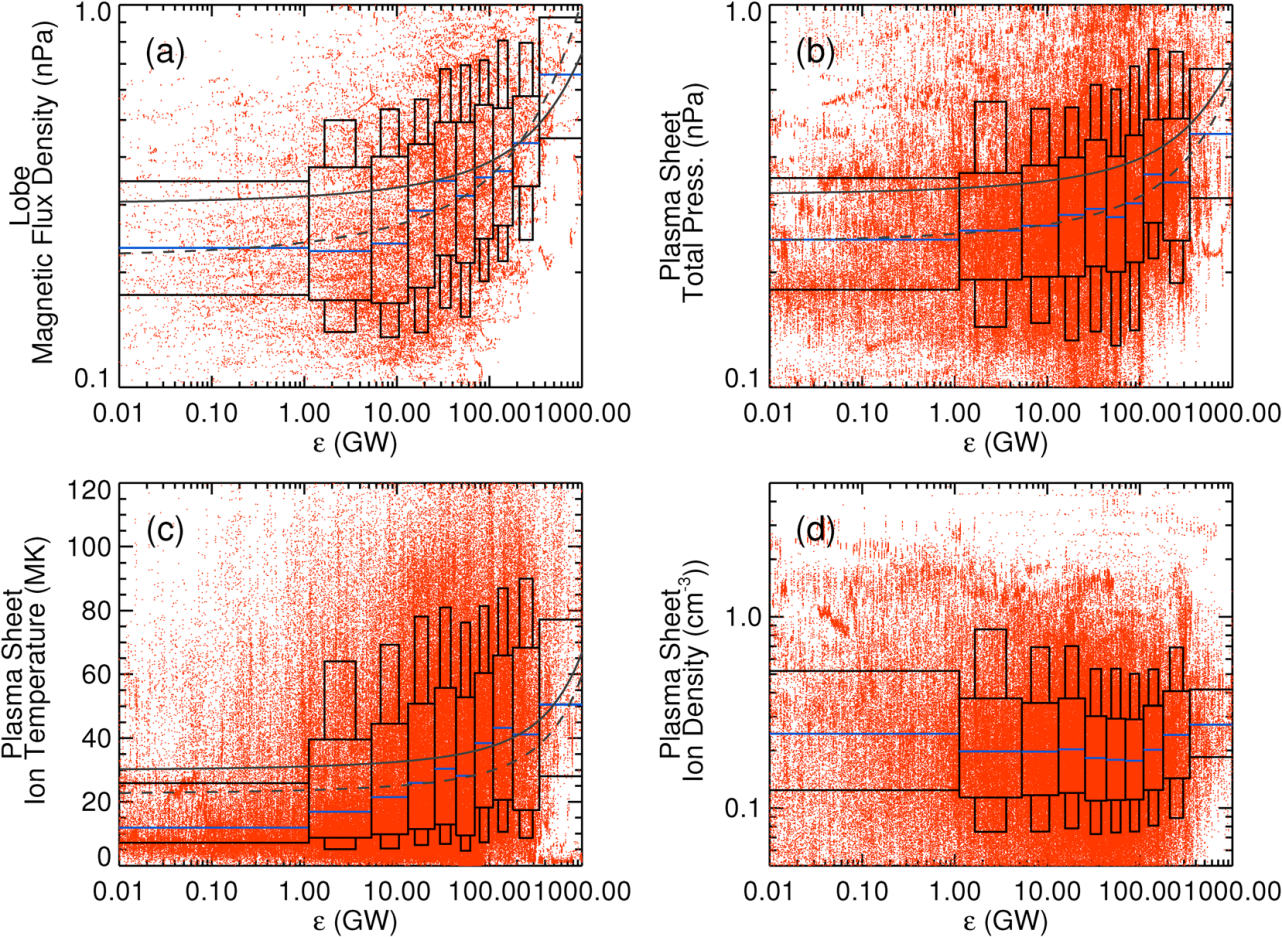
Plot of magnetotail properties against solar wind driving: (a) Magnetic pressure in the lobes; (b) total pressure in the plasma sheet (magnetic pressure + H^+^ + O^+^); (c) plasma sheet ion temperature; and (d) plasma sheet ion density. The overlaid boxes show the median (blue line), upper and lower quartiles (large box) and upper and lower deciles (small box) of the ordinate data split into deciles of the solar wind driving from the entire data set. The grey lines show the fits to our semiempirical model. The solid lines show fits of these models to the whole data set, and the dashed lines show fits to the shown median values.

Figure 2 shows the (a) magnetic pressure in the lobes, (b) total pressure in the plasma sheet, taken to be the sum of the ion (H^+^ + O^+^) pressure and magnetic pressure, (c) ion temperature, and (d) ion density in the plasma sheet against the 15 min averaged *ε* function for growth phase intervals. The overlaid boxes indicate the distribution of data points in deciles of the complete solar wind data set (rather than the subset of solar wind data cotemporaneous with the Cluster data set). The boxes show the medians (blue line), upper and lower quartiles (thick boxes) and upper and lower deciles (thin boxes). Since the binned Cluster data are not always well described by a symmetric normal distribution, we have chosen this method of display to highlight the characteristics of the data points that would be lost if the data were expressed as a mean and standard deviation.

The magnetic pressure in the lobes (Figure 2a) and total pressure in the plasma sheet (Figure 2b) increase with increased solar wind driving. The Spearman's rank order correlation coefficients (*ρ*) of the entire data sets were 0.35 and 0.21, respectively, although correlating the shown medians gave *ρ* of 0.94 and 0.96, respectively. This general trend in the data supports the canonical substorm model: increased driving leads to increased magnetopause reconnection and loading of open magnetic flux into the magnetotail lobes, leading to increased flaring and increased pressure in the magnetotail. In a recent study, *Liu et al.* [2013] showed that plasma sheet pressure was moderately correlated with solar wind ram pressure. We find only a 2% correlation between the time-averaged solar wind ram pressure and the *ε* function; thus, they are effectively independent. Pressure variations during the substorm may be related to solar wind driving, while solar wind pressure defines the initial pressure within the system, resulting in a spread of observed pressures.

Figures 2a and 2b show a deviation from the lobes and plasma sheet being in pressure balance (at least statistically) under high solar wind driving. This discrepancy may be instrumental, arising from an underestimation of the ion pressure due to the ion temperature approaching the maximum energy of the CODIF instrument; the presence of an unmeasured cold ion population [e.g., *Andre and Cully,* 2012]; or the electron pressure becoming a significant component. One may also need to account for plasma convection, such that the plasma sheet and lobes are in momentum balance. However, the general trend of increasing plasma sheet pressure with increasing magnetic pressure in the lobes is observed and the considerations of small differences in the pressures are beyond the scope of this paper.

In order to consider how the energetics of the substorm cycle relate to the solar wind driving of the magnetosphere, we break the plasma pressure down into temperature and density (Figures 2c and 2d). Figure 2c shows that plasma sheet temperature increases with solar wind driving, with *ρ* for the entire data set of 0.31 and 0.83 for the shown median values. The median temperature increases by a factor of 2.9 between the lowest and highest driving deciles. The interquartile range also increases, showing that a greater range of temperatures was observed during intervals of high solar wind driving. In contrast, there is little variation in the median or interquartile ranges of density between different levels of driving (Figure 2d), although the median density drops by 28% between the first and seventh deciles.

A simple calculation of a 40 × 3 × 30 Re plasma sheet with a density of 0.1−0.3 cm^−3^ and temperature of 20−30 MK gives a thermal energy of ∼1.57e13 J, about 10−50% of the energy ejected in plasmoids and 1.5−7% of the total energy released in a substorm [*Ieda et al.,* 1998; *Tanskanen et al.,* 2002]. As such, an increase in the plasma sheet temperature of 20 MK between intervals of low and high solar wind driving means ∼1.5e13 J extra is stored in the plasma sheet prior to substorm onset during intervals of high solar wind driving.

Given that the autocorrelation *e*-folding time of the solar wind velocity is 32 h compared to 5–10 h for the solar wind magnetic field [*Borovsky et al.,* 1998] and that the solar wind ram pressure is approximately constant during individual substorms [*Kistler et al.,* 2006], we determine a functional relationship between solar wind driving and plasma sheet pressure assuming that pressure variations in the plasma sheet during the growth phase result from the addition of magnetic flux to the tail lobes. *Morley et al.* [2007] determined a functional form of the polar cap potential with respect to solar wind driving (their equation (10)):


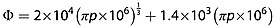
(1)

where 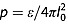 is the solar wind power per unit area. Taking this equation and assuming no loss of lobe flux during the growth phase, the increase in magnetic flux in the lobes is then


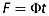
(2)

where *t* is an integration period, taken in this case to be an hour. The lobe magnetic field strength is then


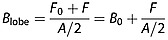
(3)

where *A* is the cross-sectional area of the magnetotail, *F*_0_ is the initial lobe flux, and *B*_0_ is the initial lobe magnetic field. We assume that the cross-sectional area of the lobe is fixed during the growth phase, assuming that there is no variation in the solar wind ram pressure during a substorm [*Kistler et al.,* 2006] and that the increase in the lobe cross-sectional area from the increase in lobe magnetic flux is negligible. Taking the magnetotail to be in pressure balance, we fit the magnetic pressure based on equation (3) to the lobe magnetic pressure and plasma sheet total pressure in Figures 2a and 2b using the MPFIT package [*Markwardt*, 2009]. The grey solid line shows the fit to all the data and the grey dashed line shows the fit to the shown medians. The fitting gives *B*_0_∼24–33 nT and *A* ∼ 23.5^2^-27.5^2^ π *R_E_*^2^ which are reasonable values for the magnetotail. The coefficients of determination (*R*^2^) for the fits to all the data were less than 0.18, indicating that the spread of the data is not dependent on the solar wind driving. For the medians, *R*^2^ is higher than 0.95, indicating that the general trends in the data are well described by the model. We note that the cross-sectional area for individual events is dependent on the solar wind ram pressure, which can vary by a factor of 2 between storm and nonstorm intervals [*Kistler et al.,* 2006]. This is not accounted for in our analysis.

We expand this model to calculated plasma sheet temperature under the assumption of pressure balance in the magnetotail,


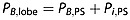
(4)


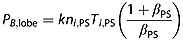
(5)

thus the temperature in the plasma sheet for a given value of *β* is given by


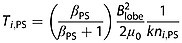
(6)

The grey lines in Figure 2c show the functional form of the plasma sheet temperature using equations (1), (3), and (6) for *β* = 0.35 and for constant density (after Figure 2d) using the *B*_0_ and *A* from fitting the plasma sheet total pressure (solid line) and the medians of the total pressure (dashed line). We note that *β*_PS_ can vary with *T*_*i*,PS_ and that a full expansion of this equation will be dependent on the plasma equations of state used.

## 4. Plasma Sheet Temperature Versus Substorm Size

**Figure 3:**
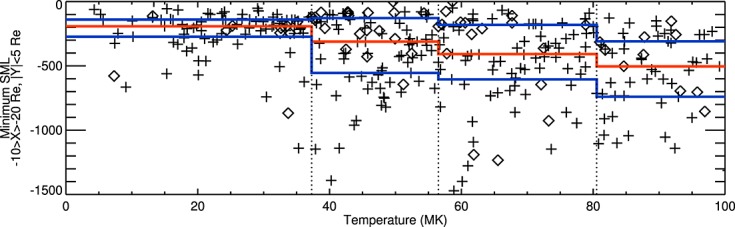
Minimum SML during a substorm expansion phase against mean plasma sheet temperature in the 5 min of growth phases prior to substorm onset. Crosses (diamonds) show data from the outer (inner) plasma sheet as defined by *Boakes et al.* [2014]. The red line shows the median SML_min_ in the four temperature quartile groups. The blue lines show the upper and lower quartiles of SML_min_.

Figure 3 compares the plasma sheet temperatures averaged over the last 5 min of a growth phase when Cluster was in the inner or outer plasma sheet with the minimum SML in the subsequent expansion phase. The median temperature in the boundary region was 15 MK compared with 54 MK and 60 MK in the outer and inner plasma sheet, respectively; thus, we do not consider events in the boundary region for this analysis. The plasma sheet temperature observations are separated into quartile groups indicated by the vertical dotted lines. In each group, the median SML is indicated with a horizontal red line, and the upper/lower quartiles of SML are indicated with horizontal blue lines.

The minimum SML (SML_min_) during the expansion phase, which can be taken as an indication of the intensity of the substorm, increases with temperature of the plasma sheet just prior to onset. The difference between the median SML_min_ between subsequent groups is statistically significant, assessed using the Mann-Whitney-Wilcoxon test, beyond the 95% level. The spread of data is large (with interquartile ranges of up to 137% of the median) and the Spearman's correlation coefficient is low (*ρ* = 0.43) but statistically significant beyond the 99% level. These results show a weak link between substorm expansion phase magnitude and the plasma sheet temperature observed by Cluster prior to onset.

## 5. Discussion

Using 9 years of magnetotail data from Cluster 4, we have shown that the plasma sheet pressure and temperature increase with solar wind power input during the substorm growth phase. Similar effects can be seen during the substorm expansion and recovery phases (not shown) but this is expected if one considers that the substorm expansion phases heats a preheated plasma sheet.

*Tsyganenko and Mukai* [2003] developed a plasma sheet model using data from Geotail that is described by a series of equations employing up to 16 terms. In this study, we have shown that a first approximation of the plasma sheet pressure and temperature can be obtained from a physical model of the magnetotail and using an empirical model of the cross polar cap potential of *Morley et al.* [2007] that is dependent on solar wind power input, assuming a constant area of the magnetotail.

Given that the magnetosphere's shape is defined by pressure balance between the solar wind and magnetospheric plasmas [e.g., *Shue et al.,* 1997], previous studies of the tail have tended to examine changes in pressure [e.g., *Kropotkin and Lui,* 1995; *Miyashita et al.,* 2009]. Under the assumption that magnetospheric plasma behaves as an ideal gas, with *P* = nkT, pressure varies with density, temperature, or both. Thermodynamically isothermal changes in pressure would result in variations in density at constant temperature, whereas adiabatic changes in pressure would result in temperature changes. Statistical studies examining the thermodynamics of the plasma sheet have concluded that the plasma sheet is predominantly thermodynamically adiabatic [e.g., *Baumjohann and Paschmann,* 1989; *Goertz and Baumjohann,* 1991], with this property being used to model and understand the convection of plasma [e.g., *Pontius and Wolf,* 1990; *Erickson*, 1992; *Birn et al.,* 1996], although nonadiabatic heating effects are present following substorm onset [*Baumjohann et al.,* 1991; *Huang et al.,* 1992]. We have shown that, on average, the plasma sheet temperature is higher during intervals of high solar wind driving, while the median plasma sheet density is approximately invariant. Our results may be interpreted as demonstrating the large-scale thermodynamically adiabatic nature of the plasma sheet, at least during the substorm growth phase, by showing that increased solar wind driving of the magnetosphere results in statistical increases in temperature at near constant density. However, in order to validate this hypothesis it is necessary to rule out the possibility that the plasma sheet is more likely to be at a higher temperature during intervals in which our solar wind driving function is large, i.e., to rule out that the higher temperatures and a large solar wind driving function have a common source. Given the multitude of correlations that have been shown between various solar wind and magnetospheric parameters, this task is nontrivial; however, by separating out the velocity and magnetic field components of the *ε* function, we find that the increase in temperature with these components is comparable.

A relationship between the solar wind velocity and temperature has previously been shown [e.g., *Burlaga and Ogilvie*, 1970; *Richardson and Smith*, 2003]. *Borovsky et al.* [1998] also showed a weak correlation (∼25%) between solar wind velocity and plasma sheet temperature with higher solar wind speeds giving higher ion temperatures in the magnetosheath. One might therefore consider that the temperature of the plasma sheet is linked to its seed population, be that the magnetosheath or solar wind. However, given that the plasma sheet ion temperature is approximately an order of magnitude greater than in the magnetosheath and 2 orders of magnitude greater than the solar wind, in order for this to be the case, the mechanisms that heat the plasma sheet must multiply the ion thermal energy rather than simply adding to it and it is unclear what processes may do this.

Case studies [*Nagai et al.,* 1997] and superposed epoch analysis [*Kistler et al.,* 2006] have shown that the plasma sheet density increases for near constant temperature during the substorm growth phase, with this effect being most prominent for substorms observed during the main phase of a storm. These results imply that the pressure changes in the plasma sheet during the growth phase are isothermal and must, by extension, result in any thermal energy added to the plasma sheet from the work done by the increasing lobe magnetic flux density being transmitted out of the magnetotail plasma, presumably into the ionosphere. Given that we have not explicitly examined temporal changes in plasma sheet temperature or density, our results are not directly comparable; however, isothermal changes in the plasma sheet would be in direct contrast to the adiabatic changes discussed above.

Our results can be interpreted as showing that thermal energy can be added to the plasma sheet during the substorm growth phase without the need for reconnection or a rapid reconfiguration of the magnetosphere. While this additional energy may be small (of the order of 1% of the total substorm energy budget), it may be significant, particularly in controlling substorm onset. The physics controlling the onset of the magnetospheric substorm are still the subject of rich debate. Both plasma instabilities [e.g., *Lui,* 2004; *Rae et al.,* 2009, 2012; *Walsh et al.,* 2010] and reconnection [e.g., *Angelopoulos et al.,* 2008; *Nishimura et al.,* 2011; *Sergeev et al.,* 2012] play important roles. As yet, we do not have a clear understanding of the processes that control the onset, rate or duration of reconnection in the magnetotail. Equally, the destabilization of the near-Earth magnetotail by plasma instabilities during expansion phase onset [*Rae et al.,* 2012] is not well-understood. The dependence of magnetic reconnection on plasma sheet temperature has not, to our knowledge, been investigated, but it is instructive to consider how some of the most promising plasma instabilities depend upon plasma sheet temperature. Modeling indicates that the magnetotail becomes unstable to ballooning instabilities and reconnection when the plasma beta is high [e.g., *Cheng and Zaharia,* 2004], although it is usually argued that this comes about from a local reduction in the magnetic field strength [*Pritchett and Coroniti,* 2013]. Higher plasma sheet temperatures prior to the onset of substorms and following high solar wind driving increase the plasma pressure and hence plasma beta in the inner plasma sheet. This, in turn, could lead to a greater probability of localized regions of the tail becoming unstable to the ballooning instability and the onset of substorms. In their review, *Lui* [2004] showed that many of the possible substorm onset instabilities have some dependence on temperature or temperature ratios. For example, the anomalous resistivity from the cross-field current instability is linearly dependent on the plasma temperature thus the increase in plasma sheet temperature with solar wind driving may provide the necessary conditions for substorm onset. Given our lack of knowledge in this area, our results indicate that the dependence of magnetotail energy transfer processes on temperature warrants further study.

Our results have shown that plasma sheet temperature increases with increased solar wind driving, and that higher plasma sheet temperatures are associated with larger substorms. This link may not be causal but simply a reflection of the correlation between lobe magnetic flux and plasma sheet temperature and the correlation between stored lobe magnetic energy and substorm size [e.g., *Morley et al.,* 2007; *Milan et al.,* 2009]. It is interesting to speculate that higher temperatures in the plasma sheet may increase the susceptibility of the magnetotail to various instabilities and that the resulting reconfiguration of the magnetosphere is dependent on how unstable the magnetotail is, which may be dependent on temperature, and thus how large a substorm is. Deconvolving the effect of having large stores of magnetic energy from the effect of plasma sheet temperature on various magnetotail instabilities will be nontrivial, but may be crucial in determining the physics behind substorms within this highly coupled system.

## 6. Conclusions

Using 9 years of Cluster tail observations, we have shown that the growth phase plasma sheet temperature is higher during intervals of higher solar wind driving. This is thermodynamically reasonable: work done on the plasma sheet by the increasing magnetic pressure in the lobes increases the internal energy of the plasma sheet; in an thermodynamically adiabatic magnetotail this energy cannot be easily and quickly extracted from the plasma sheet so its temperature rises. We note that we cannot fully deconvole the links between plasma sheet temperature and the components of the solar wind drivers that may increase the plasma sheet temperature through other mechanisms. Higher temperatures during intervals of high driving may increase the likelihood of the plasma sheet becoming susceptible to a number of instabilities thus increasing the likelihood of substorm onset. While the energy increase of the plasma sheet may be small compared to the total energy budget of a substorm, the thermodynamic processes and energization of the plasma sheet prior to substorm onset may be key determining onset times and substorm intensity.

## Key Points

- Growth phase plasma sheet temperature varies with solar wind driving
- Higher temperatures observed in intervals of higher driving
- Plasma sheet temperature can influence substorm onset and size
